# Big data from small tissues: extraction of high-quality RNA for RNA-sequencing from different oilseed *Brassica* seed tissues during seed development

**DOI:** 10.1186/s13007-020-00626-0

**Published:** 2020-06-05

**Authors:** Laura Siles, Peter Eastmond, Smita Kurup

**Affiliations:** Department of Plant Sciences, Rothamsted Research, Harpenden, Hertfordshire AL5 2JQ UK

**Keywords:** RNA extraction, Plant, Difficult tissue, Seed, Embryo, Endosperm, Reproductive, Seed coat, *Brassica*

## Abstract

**Background:**

Obtaining high-quality RNA for gene expression analyses from different seed tissues is challenging due to the presence of various contaminants, such as polyphenols, polysaccharides and lipids which interfere with RNA extraction methods. At present, the available protocols for extracting RNA from seeds require high amounts of tissue and are mainly focused on extracting RNA from whole seeds. However, extracting RNA at the tissue level enables more detailed studies regarding tissue specific transcriptomes during seed development.

**Results:**

Seeds from heart stage embryo to mature developmental stages of *Brassica napus* and *B. oleracea* were sampled for isolation of the embryo, endosperm and seed coat tissues. Ovules and ovary wall tissue were also collected from pre-fertilized buds. Subsequent to testing several RNA extraction methods, modifications applied to E.Z.N.A. Plant RNA and Picopure RNA Isolation kit extraction methods resulted in RNA with high yield and quality. Furthermore, the use of polyvinylpolypyrrolidone for seed coats and endosperm at green stages resulted in high-quality RNA. As a result of the introduced modifications to established RNA extraction methods, the RNA from all the above-mentioned tissues presented clear 28S and 18S bands and high RIN values, ranging from 7.0 to 10.0. The protocols reported in this study are not only suitable for different and challenging seed tissue types, but also enable the extraction of high-quality RNA using only 2 to 3 mg of starting tissue.

**Conclusions:**

Here, we present efficient, reproducible and reliable high-quality RNA extraction methods for diverse oilseed *Brassica* spp reproductive tissue types including pre-fertilization and developing seed tissues for diploid and polyploid species. The high-quality RNA obtained is suitable for RNA-Sequencing and subsequent gene expression analysis.

## Background

Seeds are the most important agricultural product, accounting for at least 70% of the world’s food supply [1]. Seeds are good sources of carbohydrates, protein and oil reserves. They can be used not only for production of vegetable oil, margarines, biodiesels, plastics and lubricants; but also, for animal and human consumption. Seeds are complex structures formed by three tissue types (embryo, endosperm and seed coat) derived from different ontogenetic origins [2] that are subject to changes in the abundance of transcripts from fertilization to seed maturity. Since the availability of Next Generation Sequencing (NGS) methods, we have the potential to measure the level of transcripts with high-quality and accuracy. With the reduction in costs for NGS methods, we can now obtain large amounts of sequence data for a large number of samples in a very cost-effective manner, providing more comprehensive spatial and temporal studies. NGS methodologies have improved over the years, allowing for RNA-Sequencing library preparations from small amounts of RNA. Hence, more studies are focusing on specific tissues in different phases of seed development or in different regions in specific developmental stages [3–7]. However, to the best of our knowledge, there is only one study in *Arabidopsis thaliana* that has investigated seed development at a high level of detail, focusing on different tissue types and subregions during seed development [8]. This is also the case for *Brassica* spp, where studies have focused only on the whole seed, specific embryo stages or the late phases of seed development, mainly related to oil biosynthesis and storage seed reserves [9–14].

Oilseed rape is a crucial source of vegetable oil and protein world-wide, being a crop with high economic importance. Extracting high-quality RNA from *Brassica* spp seeds is challenging as they are rich in polyphenols, polysaccharides, proteins and lipids which interfere with or degrade the RNA during RNA extraction procedures. Polyphenols limit RNA extraction as they bind to nucleic acids [15], and polysaccharides co-precipitate with RNA [16]. Removing these contaminants is a crucial step during RNA extraction protocols, as RNA with high purity and integrity is critical for gene expression analyses. Although there are several methods available for seed RNA extraction [17–20]; fewer protocols are available for the extraction of good quality RNA from specific seed tissue types, especially for the seed coat [9, 21–23]. Moreover, the main constraint of these protocols is the large amount of sample required, ranging between 50 and 200 mg of tissue, in order to obtain the quality and concentration of RNA required for downstream analyses.

Embryos from the Brassicaceae family follow a well-studied developmental timeline, from globular to heart, torpedo, green and mature embryo seed stages [24]. Herein, using only 2 to 3 mg of seed coat and green stage endosperm (GSEnd) ground tissue and a small number of embryos, we describe efficient and reproducible methods for isolating high-quality RNA from different tissue types from *Brassica napus* and *B. oleracea* during seed development for subsequent RNA-Sequencing. Protocols for extracting RNA from different seed tissue types are not only essential to identify and study tissue-specific genes, but also to understand how genes or cellular processes interact among different tissue types. Essentially, these protocols enable an integrated and better understanding of seed development. Moreover, the implementation of these methodologies not only reduces the hours of labour-intensive dissections, but also enables more detailed studies characterizing different seed tissues types and the prediction of gene regulatory networks across seed development.

## Methods

### Plant growth conditions and tissue type collection

**Fig. 1:**
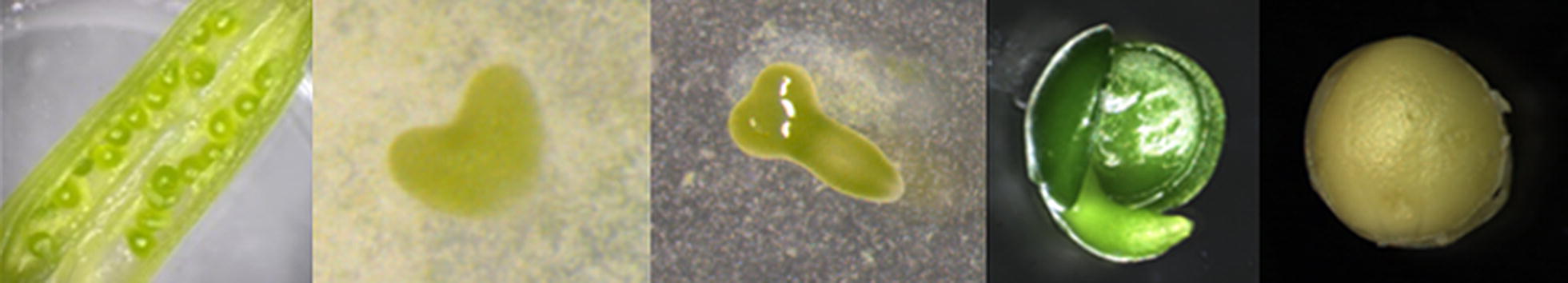
Developmental stages in *Brassica napus* and *B. oleracea*. From left to right: ovary wall and ovules, heart stage embryo, torpedo stage embryo, green stage embryo and mature stage embryo

*Brassica* plants were grown under controlled environment conditions with a 16 h photoperiod at 18 °C and 15 °C day and night temperatures respectively, under 400 W HQI lighting (225 µmol m^¯2^s^¯1^). The relative humidity was maintained at 70% day and night. Perforated bread bags (150 mm × 700 mm, WR Wright & Sons Ltd, Liverpool, UK) were used to enclose inflorescences to prevent cross-pollination from neighbouring plants before the plants initiated flowering. Flowers were manually pollinated, and developing seeds were collected at heart, torpedo, green and mature embryo stages. Flowers were tagged when pollinated to determine the developmental stage of the seed, in addition the embryos within the seed were visually checked to ensure they were at the correct stage of development prior to sampling. Buds were collected 24 h before anthesis to obtain ‘pre-fertilization stage’ ovules and ovary wall tissue (Fig. 1). Buds and pods at the desired stage of development were cut from the plant using sterile scissors. Then, they were placed in sterile water in a sterile 15 or 50 ml plastic centrifuge tube for transport to the laboratory. All dissections were carried out on a clean, sterile glass slide placed over a sterile tile on a bed of ice (dry ice for heart stage embryo, HSE) in a 14 cm diameter plastic petri dish to ensure samples were cold throughout the dissection procedure. All the dissections were performed with the aid of a dissecting microscope, and all the tissues types were collected in 1.5 ml microfuge tubes.

For ovules and ovary walls, first the sepals, petals and anthers were removed with the aid of forceps. Then, the style, stigma and gynophore were removed from the gynoecia using a clean and sterile razor blade. Next, the ovary wall was opened using a razor blade and the ovules were gently extracted by sliding the forceps through the ovary wall. After ovule extraction, all the remaining material was considered the ovary wall. The ovules and the ovary walls were transferred to clean 1.5 ml microfuge tubes using forceps.

Embryos, seed coats and endosperm tissue were separated from each other for every developmental stage except for the mature stage, in which the endosperm and the seed coat were maintained together as the endosperm is formed by a thin and fragile layer closely attached to the seed coat in this species. For the heart and torpedo stages, the embryos were aspirated with the help of 1.5 µl of RB buffer from the E.Z.N.A. Plant RNA kit (Omega Bio-tek Inc., Norcross, Georgia, USA) for each embryo using a P10 pipette for transfer to a 1.5 ml microfuge tube. During *Brassica* spp seed development, the endosperm goes through a transition from syncytial to cellular phase. When the embryo is at the heart stage, the endosperm is in the liquid syncytial phase; at the torpedo stage the endosperm starts to cellularize, finally forming a single layer of cellularized endosperm by the green embryo stage [25]. For heart stage endosperm (HSEnd) collection, the seed was held gently with a pair of forceps. Next the seed coat was perforated carefully with the aid of forceps or syringe, and the endosperm was collected using a P10 pipette while gently squeezing the embryo (a movie file shows this procedure [see Additional file 1: Video S1]). At the torpedo stage, the seed was cut open using forceps, and 1 µl of RB buffer was added to facilitate the removal of the endosperm with a pair of forceps. At the green stage the drop of buffer was omitted, and the single layer of endosperm was transferred using a pair of forceps to a 1.5 ml microfuge tube (see Additional file 2: Figure S1 for dissected seed tissue types at green stage). For mature stage, the seed was cut using a razor blade. Next, the embryo was gently scooped out with forceps, avoiding damage to the seed coat and embryo. For all stages, seed coats were transferred to a new 1.5 ml microfuge tube using forceps.

All the samples were dissected and immediately frozen in liquid nitrogen and stored at −80 °C for total RNA isolation. However, a slight modification was applied for ovules, heart and torpedo stage embryos (TSE). These samples were dissected, collected in 150 µl of RB buffer at room temperature and ground with the help of a micro-pestle in the microfuge tube before freezing in liquid nitrogen and stored at −80 °C. Different tubes with RB buffer were used in order to avoid having the dissected tissues in buffer at room temperature for long periods of time. This methodology allowed the collection and grinding of small tissues without degradation of the RNA. Ovary walls, seed coats from all developmental stages, GSEnd, and green and mature stage embryos (GSE and MSE, respectively) were finely powdered by grinding in liquid nitrogen in a mortar and pestle. For HSEnd and torpedo stage endosperm (TSEnd), no grinding was performed as the endosperm is primarily liquid at both these stages of seed development.

Based on the tissue type of interest and the developmental stage of the seed, varying amounts of starting seed material may be required. For smaller embryos as in the heart stage, approximately 100 embryos were required for efficient and high-quality RNA extractions. On the other hand, when the embryos were larger, as in torpedo, green and mature stages, only 50 embryos were needed. See Table 1 as a guideline for tissue amounts required at different developmental stages for the extraction of high-quality RNA.

**Table 1:** Amount of ground tissue used for RNA extractions and range of RNA Integrity Number (RIN) obtained for different *Brassica napus* and *B. oleracea* tissue types, n = 15

Tissue type	Amount of tissue	RIN
Ovules	6 mg	8.0–10
Ovary wall	20 mg	9.9–10
Heart stage embryo (HSE)	20–25 µl^*^	8.0–9.4
Heart stage endosperm (HSEnd)	100 endosperms	7.3–9.4
Heart stage seed coat (HSSC)	2–3 mg	7.0–9.4
Torpedo stage embryo (TSE)	10–15 µl^a^	7.7–9.3
Torpedo stage endosperm (TSEnd)	50 endosperms	7.0–8.4
Torpedo stage seed coat (TSSC)	2–3 mg	7.0–8.4
Green stage embryo (GSE)	50 mg	9.9–10
Green stage endosperm (GSEnd)	2–3 mg	7.8–9.5
Green stage seed coat (GSSC)	2–3 mg	8.0–9.2
Mature stage embryo (MSE)	50 mg	9.7–10
Mature stage seed coat (MSSC)	2–3 mg	7.7–9.3

^a^ = 100 HSE were ground in 250 µl of RB buffer from E.Z.N.A. Plant RNA kit and an aliquot of 20–25 µl was used for RNA extraction. An aliquot of 10–15 µl was used for RNA extraction of 50 TSE

### RNA extraction methods

The RNA from ovules, ovary walls, GSE, MSE, HSEnd and TSEnd were extracted following the standard E.Z.N.A. Plant RNA protocol, including the DNase I (Omega Bio-tek Inc., Norcross, Georgia, USA) digestion on column protocol. Nevertheless, some modifications in the amount of RB buffer and the final elution volume were required depending on the tissue type for obtaining high-quality RNA. For ovary walls, and for GSEand MSE, a ratio of 1:12.5 and 1:5 of RB buffer was used. Next, the standard E.Z.N.A. Plant RNA protocol was followed, eluting the RNA in 20 µl and 30 µl of diethylpyrocarbonate (DEPC) water, respectively. The protocol for HSEnd or TSEnd and ovules was slightly modified. For HSEnd and TSEnd, 250 µl of RB buffer with 5 µl of β-mercaptoethanol (β-ME) was added to the collected tissue, and the tube was vortexed vigorously for 2 min allowing the sample to thaw in the RB buffer. For ovules, 100 µl of RB buffer with 5 µl of β-ME was added to the sample to obtain a final volume of 250 µl, before vortexing for 2 min. The standard E.Z.N.A. Plant RNA kit protocol was then followed, with the elution of RNA in a final volume of 30 µl of DEPC water.

TRIzol reagent (ThermoFisher Scientific, Waltham, USA) was used for the extraction of green stage seed coat (GSSC) RNA. 1 ml of TRIzol reagent was added to 50 mg of ground GSSC tissue. The tube was vortexed vigorously for 2 min to allow the sample to thaw in the TRIzol reagent. The homogenate was then centrifuged for 5 min at 12,000*g* at 4 °C. After an incubation at room temperature for 5 min, the supernatant was removed and transferred to a new clean microfuge tube. An aliquot of 0.2 ml of chloroform was added per 1 ml of TRIzol reagent. The microfuge tube was vigorously shaken for 15 s and incubated for 2 min at 30 °C, followed by a centrifugation for 15 min at 12,000*g* at 4 °C. The upper phase of the partitioned sample was transferred to a new microfuge tube. Then, 0.25 ml of isopropanol was added, mixing the solution by tilting the tube up and down. Next, the sample was centrifuged for 8 min at 12,000*g* at 4 °C. The supernatant was discarded. The pellet was washed with 1 ml of 75% ethanol and the sample was mixed by vortexing, followed by a centrifugation for 5 min of 7,500*g* to sediment the pellet. Excess ethanol was removed, the pellet was air dried for 10 min and finally resuspended in 25 µl of RNAse free water (ThermoFisher Scientific, Waltham. USA). For mature stage seed coat (MSSC) RNA, 100 mg of ground tissue was used following the hot borate protocol combined with RNeasy Plant Mini Kit (Qiagen, Hilden, Germany) as described in [26], eluting the RNA in 30 µl of RNAse free water.

For HSE and TSE, seed coats at all developmental stages and GSEnd, the modifications applied to the Picopure RNA Isolation Kit (ThermoFisher Scientific, Waltham. USA) protocol described here allowed the extraction of high-quality RNA, with the added benefit of starting with small amounts of seed coat and green endosperm tissue. For seed coats and GSEnd, between 2 and 3 mg of ground tissue was used for RNA extraction. A ratio of 1:2 and 1:3 (v/v) polyvinylpolypyrrolidone (PVPP, ~ 110 µm particle size, Sigma-Aldrich, Saint Louis, USA) to ground tissue was used, respectively. A 50 µl aliquot of extraction buffer from the Picopure RNA Isolation kit was added, and the samples were homogenized by vortexing for 2 min. Next the samples were incubated for 30 min at 42 °C followed by a centrifugation at 2,000*g* for 2 min to allow the separation of debris and PVPP from the cell extract. This is a critical step, as any impurity can block the column, interfering with the extraction of RNA. Subsequent steps followed the protocol described in the Picopure RNA Isolation kit manual, but for clarity the next steps of the protocol are detailed here: the supernatant was transferred to a new 1.5 ml microfuge tube, and a 50 µl aliquot of 70% ethanol was added and mixed well by pipetting a few times. The cell extract and ethanol mixture was then transferred to a pre-conditioned RNA purification column (incubated for 5 min with 250 µl of conditioning buffer at room temperature and centrifuged for 1 min at 16,000*g*). The samples were centrifuged for 2 min at 100*g*, immediately followed by a centrifugation at 16,000*g* for 30 s. An aliquot of 100 µl of wash buffer (W1) was added to the purification column followed by a centrifugation of 1 min at 8,000*g*. Another 100 µl aliquot of wash buffer (W2) was added into the purification column followed by a centrifugation of 1 min at 8,000*g*. A final wash with 100 µl of wash buffer (W2) was performed by centrifugation for 2 min at 16,000*g*. If there was some residual wash buffer in the column, a re-centrifugation for 1 min at 16,000*g* was carried out. After the three washes, the purification column was transferred to a new 1.5 ml microfuge tube. An aliquot of 11 µl of elution buffer was pipetted onto the membrane of the purification column, followed by an incubation of 1 min at room temperature. The purification columns were centrifugated for 1 min at 1,000*g* followed by a final centrifugation for 1 min at 16,000*g* to elute the RNA. RNA from three technical replicates was extracted for each sample, combined and treated for DNAse treatments using the TURBO DNA-free kit (ThermoFisher Scientific, Waltham, USA). The same RNA extraction protocol was followed for HSE and TSE. However, no PVPP was added for these tissues. As HSE and TSE were already ground at the time of tissue collection, aliquots of 20–25 µl for HSE and 10–15 µl for TSE were used directly for RNA extraction. Then, the Picopure RNA Isolation kit protocol was followed as described above.

### Determination of RNA quality

The concentration and quality of the RNA was assessed spectrophotometrically at 230, 260 and 280 nm (NanoDrop 1000, LabTech, Heathfield, UK). RNA quality and integrity were evaluated by electrophoresis on 1% agarose gel and the RNA integrity number (RIN) was determined using an Agilent 2100 Bioanalyzer (Agilent Technologies, Inc.).

## Results

Different RNA extraction protocols are optimal for different tissue types. By using E.Z.N.A. Plant RNA kit and modifying the amount of ground tissue, the RB buffer ratios as well as the final elution volume, RNA of high-quality and yield was obtained from ovules, ovary walls, GSE or MSE and HSEnd and TSEnd (Table 2). However, different methods were tested to extract RNA from HSE or TSE, seed coats and GSend as there are several difficulties encountered whilst extracting RNA from these tissues. Embryos have a high transcriptional activity, but their small size presents a challenge during grinding for efficient RNA extraction. On the other hand, GSEnd-formed by a residual single layer of cellularized endosperm-and seed coats are transcriptionally less active and contain a lot of contaminants which interfere with RNA extractions. Standard RNA extractions methods using E.Z.N.A. Plant RNA kit, hot borate combined with RNeasy Plant Mini Kit or TRIzol reagent-used for tissues with high lipid content-failed to extract high-quality RNA when attempting to extract RNA from these tissues (Table 3. See Additional file 3: Figure S2 for RNA integrity results after DNAse treatment for the tissue types and RNA extraction methods shown in Table 3).

**Table 2:** Range of RNA concentration, A260/280 and A260/230 ratios for *B. napus* and *B. oleracea* tissue types extracted with the E.Z.N.A Plant RNA kit, n=15

Tissue	RNA concentration (ng/µl)	A260/280	A260/230
Ovules	158.8–382.7	2.10–2.19	1.80–2.20
Ovary wall	380.2–827.5	2.17–2.21	1.88–2.21
Green stage embryo (GSE)	749.8–1702.2	2.19–2.22	1.70–2.24
Mature stage embryo (MSE)	127.3–639.9	2.15–2.21	1.94–2.26
Heart stage endosperm (HSEnd)	108.8–637.9	2.17–2.20	1.96–2.20
Torpedo stage endosperm (TSEnd)	102.0–243.4	2.18–2.21	1.80–2.16

**Table 3:** RNA concentration and purity of the RNA obtained from different RNA extraction methods for different *B. napus* and *B. oleracea* tissue types

RNA extraction method	Tissue	RNA concentration (ng/µl)	A260/280	A260/230
E.Z.N.A. Plant RNA kit	Green stage seed coat (GSSC)	32.66	1.67	0.30
TRIzol Reagent	Green stage seed coat (GSSC)	1.57	0.73	0.43
Picopure RNA Isolation kit	Green stage seed coat (GSSC)	113.55	2.03	1.42
E.Z.N.A. Plant RNA kit	Green stage endosperm (GSEnd)	7.79	1.39	0.14
Picopure RNA Isolation kit	Green stage endosperm (GSEnd)	54.64	1.80	0.58
Picopure RNA Isolation kit	Green stage endosperm (GSEnd) + PVPP	147.57	2.11	1.74
Hot borate	Mature stage seed coat (MSSC)	24.15	1.62	0.47
Picopure RNA Isolation kit	Mature stage seed coat (MSSC)	53.86	2.11	0.53
Picopure RNA Isolation kit	Mature stage seed coat (MSSC) + PVPP	58.27	2.02	1.59
E.Z.N.A. Plant RNA kit	Heart stage embryo (HSE)	16.16	2.30	0.10
Picopure RNA Isolation kit	Heart stage embryo (HSE)	104.10	2.07	1.97
E.Z.N.A. Plant RNA kit	Torpedo stage embryo (TSE)	91.20	2.15	2.14
Picopure RNA Isolation kit	Torpedo stage embryo (TSE)	218.14	2.13	2.09

Several methods were tested for different tissues. The RNA extraction for GSSC and GSEnd using E.Z.N.A. Plant RNA kit for 50 mg of ground tissue resulted in low A260/280 and A260/230 ratios for both tissues, indicating the presence of contaminants in the samples and low RNA yield for green endosperm. RNA from 100 HSE and 50 TSE was also extracted using E.Z.N.A. Plant RNA kit. For these tissues, the RNA purity was high, but the A260/230 ratio and the RNA concentration were low for HSE but not for TSE. The use of the TRIzol reagent method for GSSC using 50 mg of ground tissue resulted in low yields and low A260/280 and A260/230 ratios. The hot borate protocol was also tested with 100 mg of MSSC ground tissue. Although proteinase K was added for the removal of proteins, the RNA obtained presented high levels of polyphenols and polysaccharides contaminants as shown by the low A260/230 ratios. The main constraints of these methods are the high amount of tissue needed for obtaining sufficient high-quality RNA.

**Fig. 2:**
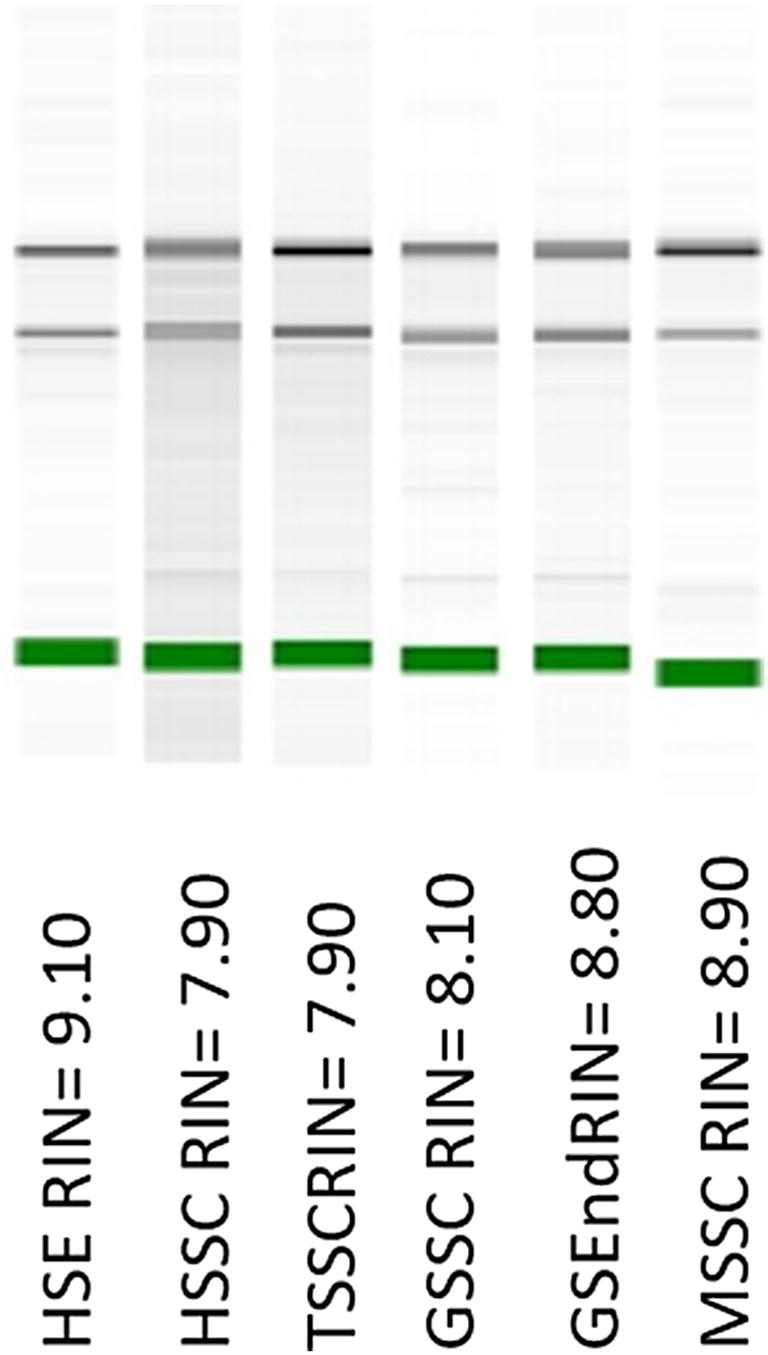
Electropherogram of the RNA isolated from the most challenging seed tissue types. RIN values are shown. *HSE* heart stage embryo, *HSSC* heart stage seed coat, *TSSC* torpedo stage seed coat, *GSSC* green stage seed coat, *GSEnd* green stage endosperm, *MSSC* mature stage seed coat

The use of the Picopure RNA Isolation kit resulted in better quality RNA for all these tissues. By using aliquots of HSE and TSE, the RNA concentration as well as the A260/280 and A260/230 ratios increased significantly, especially for HSE. For GSEnd and MSSC, using between 2 and 3 mg of ground tissue, higher RNA concentrations and A260/280 and A260/230 ratios were obtained. Due to higher concentration and quality of RNA as well as the small amount of starting material required, the Picopure RNA Isolation kit is a better extraction method for these tissues. This method also worked well for heart and torpedo stages seed coat (HSSC and TSSC, respectively). In spite of the improved quality of the RNA extracted using Picopure RNA Isolation kit, some contaminants were still present in the RNA samples, as indicated by low A260/230 ratios for green endosperm and seed coats. The addition of PVPP reduced the amount of polyphenols significantly in the extracted RNA from these tissues, increasing the A260/230 ratio from 0.2 to 1.2, and for some samples, to 1.8 (Table 3). The resulting yields of total RNA after the DNAse treatment and after using three replicates for the tissues extracted from Picopure RNA Isolation kit were high, as well as the A260/280 and A260/230 ratios, confirming the lack (or low) polysaccharide and protein contamination. Although the A260/230 ratios for GSEnd and seed coat tissues were still low, the integrity of the RNA was confirmed observing clear 28S and 18S ribosomal RNA bands with no RNA degradation. In addition high RIN values from 7 to 10 (Fig. 2, Table 1), confirmed the high-quality of the RNA obtained from different *Brassica* spp seed tissue types.

## Discussion

Obtaining high-quality RNA from different seed tissues can be challenging, especially for tissue types that do not have high transcriptional activity and may contain secondary metabolites as contaminants that interfere with RNA extraction methods. Several protocols were attempted to extract good quality RNA from these complex tissues. The TRIzol reagent allows the precipitation of RNA, DNA and proteins from a single sample. However, the resulting yield obtained from 50 mg of GSSC was unexpectedly low. Although the use of the chaotropic agent guanidium isothiocyanate for cell lysis and denaturation of ribonucleases, chloroform to separate RNA from DNA and proteins and isopropanol for RNA precipitation was attempted, the RNA obtained from GSSC using this methodology presented high level of contaminants, as shown by the low A260/280 and A260/230 ratios. For other seed species with high levels of polyphenols, RNA obtained using TRIzol reagent was also degraded and presented low A260/230 ratios [18, 19], suggesting that TRIzol is not a good methodology to extract RNA from seeds or seed coats which contain these secondary metabolites. Although hot borate has been described as a good protocol for extracting RNA from samples rich in polysaccharides, phenolic compounds and oil [27, 28], the RNA extracted from MSSC with this method was not devoid of contaminants. MSSC accumulate high amounts of polyphenols in the maturation stage of seed development, specially oxidised procyanidins which confer the dark brown colour of the seed coat characteristic of this stage of development [22, 29]. The low A260/280 and A260/230 ratios obtained from MSSC reflect protein, polyphenols and polysaccharides contamination. A limitation of the amount of tissue used or the use of proteinase K in this methodology instead of β-ME could be an alternative explanation for the high level of contaminants present in the RNA. Several methods for RNA extraction from whole seeds use PVPP for the removal of polysaccharides, proteins and polyphenol compounds [17, 19, 30]. Here, we confirm that the addition of PVPP to seed coats and GSEnd tissues significantly improved the quality of the extracted RNA by using a ratio of 1:2 or 1:3 of PVPP and ground tissue (v/v). The addition of PVPP in these tissues is important, as in these stages of development *Brassica* spp seeds accumulate polyphenols in the seed coats and polysaccharides in the endosperm. As the 1:2 or 1:3 ratio (v/v) worked for *Brassica* spp seed tissues, the amount of ground tissue needed as well as the PVPP and ground tissue ratio may vary whilst extracting RNA from other species.

For other oilseeds, such as *Sesamum indicum*, *Zea mays*, *Helianthus annuus*, *Linum usitatissimum* [31], *Arachis hypogaea* [32], *Jatropha curcas* [33] and *Chamaerops humilis* [34], different protocols use a mixture of Tris buffer combined with chloroform:isoamyl alcohol and phenol: chloroform reagents to extract RNA from whole seeds. These methodologies require high amounts of material, from 70 mg to 2 grams. However, the RNA extraction method reported here for HSE, TSE, HSSC, TSSC, GSSC, GSEnd and MSSC using Picopure RNA Isolation kit is a robust and reproducible protocol for obtaining high-quality RNA for gene expression analyses by only using 2 to 3 mg of tissue and a low number of embryos without the necessity of performing laser microdissections. Also, the use of β-ME as a reducing agent for denaturing ribonucleases that are released during tissue disruption and homogenization avoided the use of proteinase K, and pure RNA with no signs of degradation was retrieved. The modifications reported here for the E.Z.N.A. Plant RNA kit resulted in high yields and pure RNA for more transcriptionally active tissues (ovary wall, ovules, HSEnd, TSEnd, GSE and MSE) that contain high levels of polysaccharides and lipids. As described by [35], RIN values of 10 indicate completely intact RNA whereas RIN values of 1 indicate totally degraded RNA. The range of high levels of RIN values obtained for different seed tissue types during *Brassica* spp seed development confirmed the high-quality of the extracted RNA using these extraction protocols, being suitable for RNA-Sequencing. The modifications applied to E.Z.N.A. Plant RNA kit and Picopure RNA Isolation kit extraction methods described were successfully used for different *Brassica* spp genotypes including the diploid and polypoid species, obtaining a minimum of 25 million reads and 7.5 GB of raw data for all the seed tissue types.

## Conclusions

Herein, we report efficient, reliable and reproducible high-quality RNA extraction methods for a wide range of oilseed *Brassica* spp tissue types. By only using between 2 and 3 mg of tissue, high-quality RNA can be extracted from the most challenging seed tissue types in different stages of seed development. These methodologies can be used for diploid and polyploid species as well as for other oilseed species and for species with procyanidins in their seed coats. The high-quality of the RNA makes it suitable for RNA-Sequencing and subsequent gene expression analysis, allowing more comprehensive studies and the prediction of gene regulatory networks across seed development.

## Supplementary information

**Additional file****1****: VideoS1.** Video demonstrating the extraction of liquid HSEnd. The extracted endosperm is shown placed on the glass slide solely for demonstration purpose. This drop would be transferred directly to the 1.5 ml microfuge tube upon confirmation of embryo stage.

**Additional file 2: Figure S1.** Seed coat, cellularized endosperm and embryo tissues separated at green stage.

**Additional file 3: Figure S2.** Gel electrophoresis for different seed tissue types and RNA extraction methods shown in Table 3 after DNase treatment. (1) 6 µl aliquot from 30 µl was loaded for GSSC RNA extracted with E.Z.N.A. Plant RNA kit. (2) 1 µl aliquot from 24 µl was loaded for GSSC RNA extracted with Picopure RNA Isolation kit. (3) 2 µl aliquot from 30 µl was loaded for GSEnd RNA extracted with Picopure RNA Isolation kit. Protein contamination is observed. (4) 1 µl aliquot from 24 µl was loaded for GSEnd RNA extracted with Picopure RNA Isolation kit and PVPP. (5) 2 µl aliquot from 24 µl was loaded for MSSC RNA extracted with Picopure RNA Isolation kit. Protein contamination is observed. (6) 2 µl aliquot from 24 µl was loaded for MSSC RNA extracted with Picopure RNA Isolation kit and PVPP. (7) 1 µl aliquot from 18 µl was loaded for HSE RNA extracted with Picopure RNA isolation kit. (8) 1 µl aliquot from 30 µl was loaded for TSE RNA extracted with E.Z.N.A. Plant RNA kit. (9) 0.5 µl aliquot from 18 µl was loaded for TSE RNA extracted with Picopure RNA Isolation kit. GSSC RNA extracted with TRIzol, GSEnd RNA extracted with E.Z.N.A. Plant RNA kit, MSSC RNA extracted with hot borate and HSE RNA extracted with E.Z.N.A. Plant RNA kit are not shown as the RNA amount extracted was not sufficient to be analysed by electrophoresis.

## Data Availability

The datasets used and/or analysed during the current study are available from the corresponding author on reasonable request.

## Abbreviations

HSE: Heart stage embryo
HSEnd: Heart stage endosperm
HSSC: Heart stage seed coat
TSE: Torpedo stage embryo
TSSC: Torpedo stage seed coat
TSEnd: Torpedo stage endosperm
GSE: Green stage embryo
GSEnd: Green stage endosperm
GSSC: Green stage seed coat
MSE: Mature stage embryo
MSSC: Mature stage seed coat
ME: Mercaptoethanol
DEPC: Diethylpyrocarbonate
