# Supplementary figures and images for: Big data from small tissues: extraction of high-quality RNA for RNA-sequencing from different oilseed Brassica seed tissues during seed development
Source: Plant Methods. 2020 Jun 5;16:80. doi: 10.1186/s13007-020-00626-0 (PMC7275424; doi:10.1186/s13007-020-00626-0)

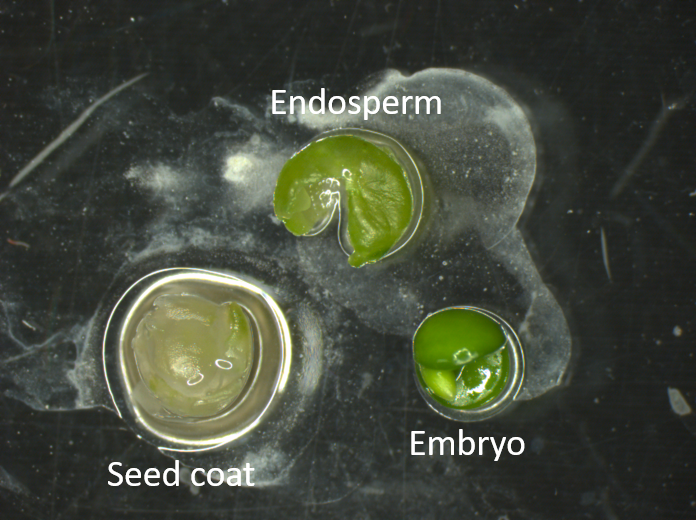

Supplement: Supplementary file 2 — Additional file 2: Figure S1. Seed coat, cellularized endosperm and embryo tissues separated at green stage. [file 13007_2020_626_MOESM2_ESM.tif]

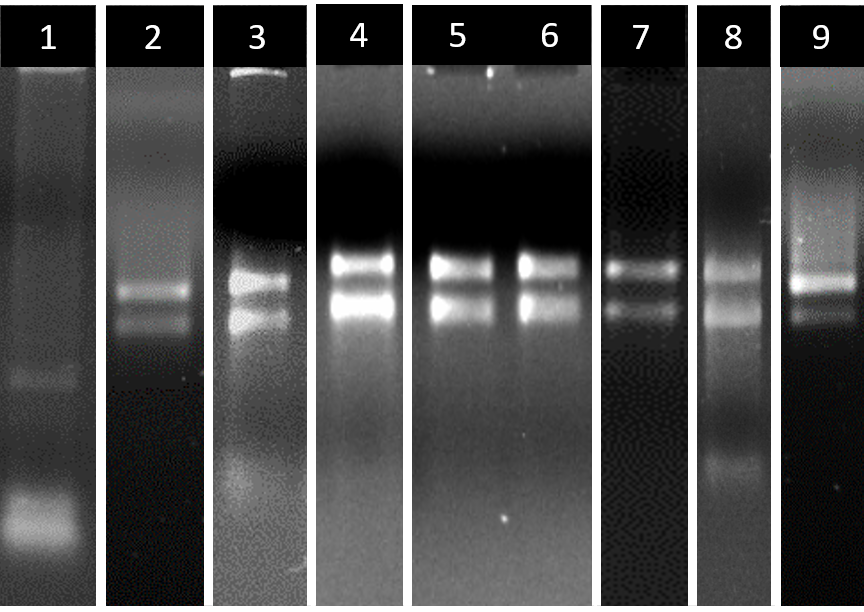

Supplement: Supplementary file 3 — Additional file 3: Figure S2. Gel electrophoresis for different seed tissue types and RNA extraction methods shown in Table 3 after DNase treatment. (1) 6 µl aliquot from 30 µl was loaded for GSSC RNA extracted with E.Z.N.A. Plant RNA kit. (2) 1 µl aliquot from 24 µl was loaded for GSSC RNA extracted with Picopure RNA Isolation kit. (3) 2 µl aliquot from 30 µl was loaded for GSEnd RNA extracted with Picopure RNA Isolation kit. Protein contamination is observed. (4) 1 µl aliquot from 24 µl was loaded for GSEnd RNA extracted with Picopure RNA Isolation kit and PVPP. (5) 2 µl aliquot from 24 µl was loaded for MSSC RNA extracted with Picopure RNA Isolation kit. Protein contamination is observed. (6) 2 µl aliquot from 24 µl was loaded for MSSC RNA extracted with Picopure RNA Isolation kit and PVPP. (7) 1 µl aliquot from 18 µl was loaded for HSE RNA extracted with Picopure RNA isolation kit. (8) 1 µl aliquot from 30 µl was loaded for TSE RNA extracted with E.Z.N.A. Plant RNA kit. (9) 0.5 µl aliquot from 18 µl was loaded for TSE RNA extracted with Picopure RNA Isolation kit. GSSC RNA extracted with TRIzol, GSEnd RNA extracted with E.Z.N.A. Plant RNA kit, MSSC RNA extracted with hot borate and HSE RNA extracted with E.Z.N.A. Plant RNA kit are not shown as the RNA amount extracted was not sufficient to be analysed by electrophoresis. [file 13007_2020_626_MOESM3_ESM.tif]
